# Hypothalamic ΔFosB prevents age-related metabolic decline and functions via SNS

**DOI:** 10.18632/aging.101157

**Published:** 2017-01-20

**Authors:** Kazusa Sato, Anna Idelevich, Kenichi Nagano, Glenn C. Rowe, Francesca Gori, Roland Baron

**Affiliations:** ^1^ Department of Medicine, Harvard Medical School and Endocrine Unit MGH, and Division of Bone and Mineral Metabolism, Department of Oral Medicine, Infection and Immunity, Harvard School of Dental Medicine, Boston, MA 02115, USA

**Keywords:** ΔFosB, glucose, hypothalamus, SNS, aging

## Abstract

The ventral hypothalamus (VHT) integrates several physiological cues to maintain glucose homeostasis and energy balance. Aging is associated with increased glucose intolerance but the underlying mechanisms responsible for age-related metabolic decline, including neuronal signaling in the VHT, remain elusive. We have shown that mice with VHT-targeted overexpression of ΔFosB, a splice variant of the AP1 transcription factor FosB, exhibit increased energy expenditure, leading to decreased adiposity. Here, we show that VHT-targeted overexpression of ΔFosB also improves glucose tolerance, increases insulin sensitivity in target organs and thereby suppresses insulin secretion. These effects are also observed by the overexpression of dominant negative JunD, demonstrating that they occur via AP1 antagonism within the VHT. Furthermore, the improved glucose tolerance and insulin sensitivity persisted in aged animals overexpressing ΔFosB in the VHT. These beneficial effects on glucose metabolism were abolished by peripheral sympathectomy and α-adrenergic, but not β-adrenergic, blockade. Taken together, our results show that antagonizing AP1 transcription activity in the VHT leads to a marked improvement in whole body glucose homeostasis via activation of the SNS, conferring protection against age-related impairment in glucose metabolism. These findings may open novel avenues for therapeutic intervention in diabetes and age-related glucose intolerance.

## INTRODUCTION

For the past few decades, obesity and its closely associated co-morbidities, such as type II diabetes mellitus have shown a steady rise among all age groups, from the adolescents to elderly. Several factors, including early diet-associated neuronal rewiring and epigenetic modifications, were suggested to lead to metabolic disturbances later in life [1–3]. While many peripheral metabolism regulating sites, such as pancreas and liver have been well studied, the central pathways, sensing glucose and affecting insulin production, feeding and energy expenditure, remain discordantly less explored. Within the brain, the hypothalamus, and its several nuclei, including the Arcuate Nucleus (ARC), Paraventricular Nucleus (PVN), Preoptic Area (POA), and others, possess multiple features implicated in the control of glucose homeostasis [4,5]. The fenestrated vasculature grants hypothalamus with direct exposure to the arterial blood carrying nutrients, as well as hormones such as insulin, leptin, ghrelin, translating these peripheral cues to adaptive physiological responses. For example, central administration of insulin provokes hyperpolarization of ARC-residing AgRP and POMC neurons [6], whereas neuron-specific ablation of insulin receptors is associated with diet-induced obesity [7], and ablation from AgRP, but not POMC neurons, alters hepatic glucose production [8,9]. Beyond hormonal cues, glucose itself appears to function not only as fuel, but also as a signaling molecule within the hypothalamus. Neurons and astroglia express high levels of GLUT2 and low affinity hexokinase, which allows them to sense glucose and the ATP/AMP ratio [10,11]. This sensing leads to a rapid cessation of feeding following intraventricular injection of glucose, via excitation or inhibition of neuronal activity [12].

Although the exact mechanisms of central regulation of glucose homeostasis are only partially elucidated, it is known that hypothalamic neurons activate the sympathetic (SNS) and parasympathetic (PNS) branches of the autonomous nervous system, which control insulin and glucagon secretion [13], as well as pancreatic β-and α-cell number [14]. Noradrenergic SNS endings are associated with insulin suppression, while PNS endings, containing mainly acetylcholine, confer an opposite effect of induced insulin secretion. Inactivation of GLUT2 from neurons, was demonstrated to lower β-cell proliferation, suppress PNS tone, and lead to an overall glucose intolerance. Moreover, the hypothalamus is known to exert SNS-dependent regulation of brown fat thermogenic activity, which is implicated in the regulation of whole body glucose metabolism [15]. This complex machinery, relying on multiple factors aimed at containing glucose within strict levels, deteriorates with aging, and with aging-related neurodegenerative disorders [16].

Interestingly, the role of aforementioned factors, including insulin and leptin, is not restricted to the regulation of glucose, but governs energy expenditure, adiposity and glucose homeostasis as a whole. In previous studies we have shown that transgenic mice expressing ΔFosB, a naturally occurring splice isoform of the AP1 transcription factor FosB, under the control of broad enolase 2 (ENO2) promoter exhibit a phenotype of increased energy metabolism, enhanced glucose handling and reduced adiposity [17–20]. ΔFosB, which lacks the C-terminal transactivation domain, behaves as an AP1 antagonist [21]. In attempt to identify the site of action, we targeted ΔFosB, or the artificial AP1 antagonist dominant-negative JunD (DNJunD), selectively to the ventral hypothalamus (VHT) of mice by means of stereotaxic viral delivery and recapitulated energy and fat phenotypes observed in ENO2-ΔFosB model [18,20]. In this study, we aimed to explore whether the effects on adiposity and energy expenditure of centrally delivered ΔFosB are: 1. Associated with changes in whole body glucose metabolism; 2. Affect glucose metabolism through the course of aging. Furthermore, we set to investigate the mechanism by which these metabolic effects are mediated. Our data demonstrate that VHT over-expression of ΔFosB improves glucose profile, and is associated with increased glucose tolerance and insulin sensitivity in the peripheral organs. These effects persist in aged animals, conferring protection against age-related decline in insulin sensitivity and increased adiposity. Finally, we show that central ΔFosB influences glucose metabolism via SNS, by a mechanism involving α-, but not β-adrenergic receptors (AR). These findings opens roadway for future search of anti-aging therapeutics.

## RESULTS

### Site-specific overexpression of ΔFosB in the ventral hypothalamus

**Figure 1:**
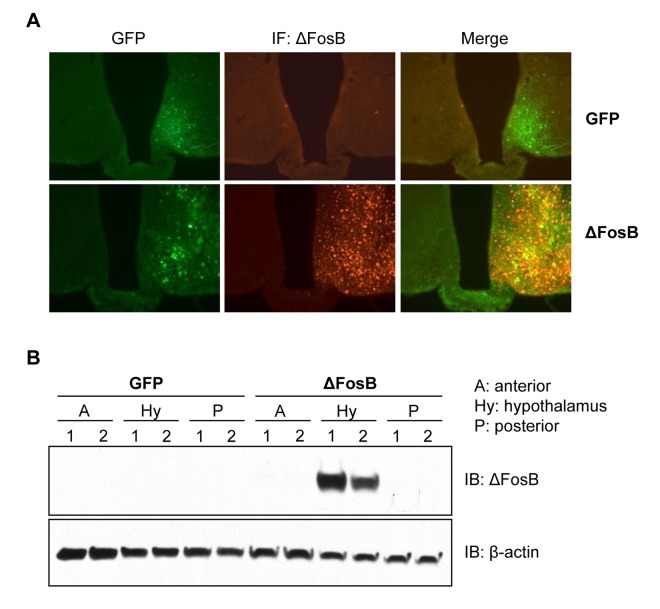
Site specific overexpression of ΔFosB in the VHT Mice received stereotaxic injections of either AAV-ΔFosB or AAV-GFP. Only right hemisphere was injected with viruses, left hemisphere was injected with saline (sham) and sacrificed 1 week post-surgically (n=3). Brain frozen sections were subjected to histological analysis (**A**) Immunofluorescent staining with anti-ΔFosB antibody followed by Alexa-fluor568 conjugated secondary antibody (red). Green fluorescence originates from ires-GFP-AAV backbone. X100 magnification. (**B**) Western blotting of gross brain sections with anti-ΔFosB using β-actin as housekeeping control. Blots of two mice per virus were shown.

To examine the effects of central ΔFosB on whole body glucose homeostasis we utilized our previously established method of stereotaxically-assisted, virally-mediated gene transfer, where AAV-ΔFosB or AAV-GFP (control) are injected bilaterally into the VHT of adult mice. To validate the specificity of the induced expression, mice received stereotaxic injections of either AAV-ΔFosB or AAV-GFP in the right hemisphere and saline in the left hemisphere (sham) (Fig. 1A). GFP expression was observed in the VHT of both AAV-GFP- and AAV-ΔFosB-injected sides, but not on the sham-injected side. ΔFosB protein expression, as assessed by immunostaining, was observed only in the AAV-ΔFosB-injected side where it co-localized with GFP fluorescence (Fig. 1A). Western blotting confirmed ΔFosB overexpression in the hypo-thalamus (Fig. 1B). Overall, these data demonstrate our ability to deliver ΔFosB specifically to the VHT.

### Overexpression of ΔFosB in the ventral hypothalamus improves glucose metabolism despite a lower insulin response to glucose

**Figure 2:**
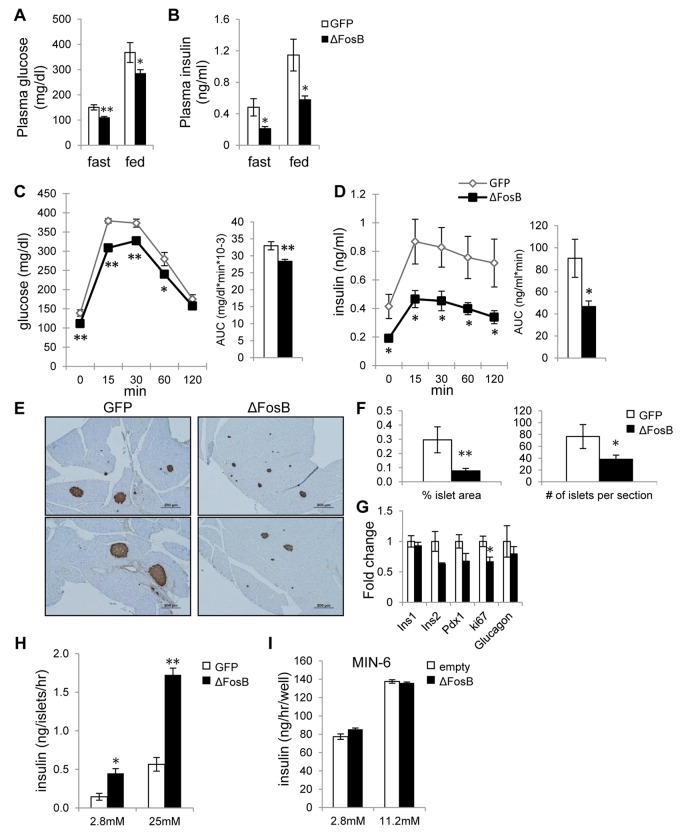
VHT overexpression of ΔFosB improves glucose profile despite lower insulin response Mice were stereotaxically injected into VHT with AAV-ΔFosB or AAV-GFP and glucose metabolism assessed 8 weeks post-surgically (n=8). (**A**) Fasted and fed glucose levels (**B**) Fasted and fed insulin levels (**C**) GTT glucose (**D**) GTT insulin (**E**) Immunostaining of pancreas with anti-insulin antibody. Scale bar; 200μm. (**F**) Histomorphometry of insulin-stained pancreatic islets (**G**). qPCR analysis of isolated pancreatic islets. (**H**) GSIS test of isolated pancreatic islets from AAV-GFP and AAV-ΔFosB mice. (**I**) GSIS test of MIN-6 cells transfected with ΔFosB. Data are presented as mean ± SEM. *p<0.05, **p<0.01.

To characterize the effects of VHT-overexpressed ΔFosB on glucose metabolism, several tests were performed. First, we measured blood glucose and insulin levels in mice injected with AAV-ΔFosB or AAV-GFP (control) at the age of 7-8 weeks and analyzed 8 weeks post-surgery. Glucose and insulin levels at both fasted and fed state were lower in the AAV-ΔFosB group compared to control (Fig. 2A,B). The glucose tolerance test (GTT) showed that AAV-ΔFosB injected mice displayed lower glucose levels following an i.p. glucose bolus than control (Fig. 2C), suggesting better glucose tolerance. Contrary to our expectations, the improved glucose tolerance was not due to increased insulin levels: the AAV-ΔFosB group revealed significantly lower insulin levels during GTT (Fig. 2D), suggesting that the observed increased glucose tolerance might be due to higher insulin sensitivity. In agreement with this hypothesis, pancreatic islets of AAV-ΔFosB mice were markedly smaller in size than those of control, as evident from insulin immunostaining and morphometric analysis (Fig. 2E,F). Examination of gene expression in isolated islets showed decreased insulin 2, Pdx-1 and Ki-67 mRNA levels in AAV-ΔFosB mice compared to AAV-GFP mice, whereas there were no changes in insulin 1 and glucagon mRNAs (Fig. 2G). Since reduction in Pdx1 expression in pancreatic β-cells has been linked with an increased rate of apoptotic cell death [32, 33], possibly explaining the small islets, we then tested whether AAV-ΔFosB islets displayed a dysfunction in glucose-stimulated insulin secretion (GSIS). Surprisingly, despite smaller islet size and reduced Pdx-1 expression, the GSIS test performed on isolated AAV-ΔFosB pancreatic islets showed a dose-dependent increase in both basal insulin level and glucose-stimulated insulin secretion compared to control (Fig. 2H). This induction of insulin secretion in islets isolated from AAV-ΔFosB mice is in contrast with the observed suppression of insulin secretion in response to glucose bolus in the same mice. This suggests that the suppressed *in vivo* insulin response due to VHT ΔFosB is driven centrally and not islet cell- autonomously.

Supporting this concept, ΔFosB overexpression in the pancreatic MIN-6 cell line failed to induce insulin (Fig. 2I). Taken together, these results support the ability of central ΔFosB to stimulate whole body glucose uptake, when overexpressed in the VHT.

### Overexpression of ΔFosB in the ventral hypothalamus increases insulin sensitivity in the periphery

**Figure 3:**
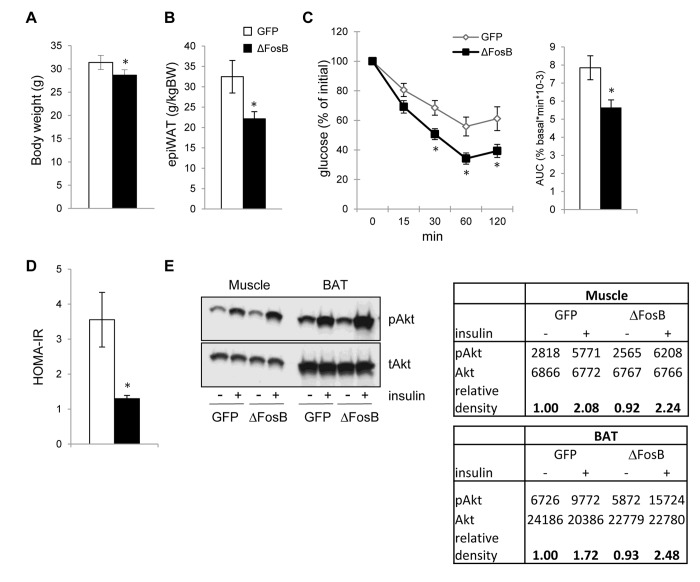
VHT overexpression of ΔFosB increases insulin sensitivity in the periphery Mice were stereotaxically injected into VHT with AAV-ΔFosB or AAV-GFP and insulin response was assessed 8 weeks post-surgically (n=8). (**A**) Body weight (**B**) Abdominal epididymal fat pad weight (**C**) ITT, data are presented as percentage of initial blood glucose concentration (**D**) HOMA-IR (**E**) Western blotting of Akt phospholylation in skeletal muscle and brown fat (BAT). Data are presented as mean ± SEM. *p<0.05, p<0.01.

Improved glucose tolerance despite a lower insulin response in AAV-ΔFosB mice suggests increased insulin sensitivity. Thus, we next examined whether VHT-ΔFosB overexpressing mice exhibit altered insulin sensitivity in peripheral tissues, in addition to the pancreatic elevated ability to secrete insulin. Confirming our earlier observations [20], body weight and visceral epididymal fat pad weight were significantly lower in the AAV-ΔFosB mice than in control mice (Fig. 3A,B). The insulin tolerance test (ITT) showed that AAV-ΔFosB mice exhibited a greater decrease in plasma glucose levels in response to insulin and a lower AUC (Fig. 3C), suggesting a higher efficacy of insulin in clearing glucose from the circulation. Accordingly, the insulin resistance index (HOMA-IR) was found to be almost two-fold lower in AAV-ΔFosB group than control (Fig. 3D), indicating higher insulin sensitivity.

Finally, providing further evidence in support of increased insulin sensitivity, we found increased relative level of phosphorylated AKT in skeletal muscle and in interscapular brown adipose tissue (BAT) in mice with VHT-ΔFosB overexpression (Fig. 3E). Taken together, these data suggest that expression of ΔFosB in the VHT increases whole body insulin sensitivity.

### The improvement of insulin sensitivity precedes the reduction in visceral adiposity

**Figure 4:**
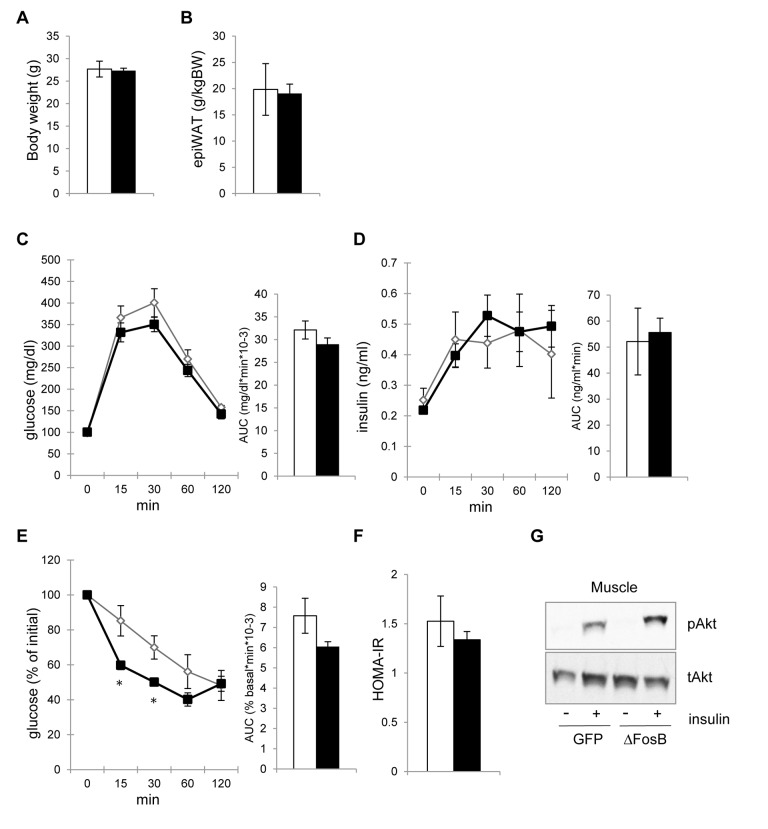
VHT-overexpression of ΔFosB-mediated amelioration of insulin sensitivity precedes reduction in visceral adiposity Mice were stereotaxically injected into VHT with AAV-ΔFosB or AAV-GFP and insulin response was assessed 2-3 weeks post-surgically (n=5). (**A**) Body weight (**B**) Abdominal epididymal fat pad weight (**C**) GTT glucose (**D**) GTT insulin (**E**) ITT glucose (**F**) HOMA-IR. (**E**) Akt phospholylation in skeletal muscle after insulin treatment. Data are mean ± SEM. *p<0.05, p<0.01.

Multiple studies outline the positive effect of weight loss and % body fat reduction on glucose metabolism in both healthy individuals and individuals with metabolic syndrome [22]. Given the smaller fat pad size in transgenic ENO2-ΔFosB [18] as well as in VHT-overexpressing AAV-ΔFosB models (Fig. 3B), we then determined whether the increase in insulin sensitivity was secondary to the reduction in visceral adiposity. For this purpose, we investigated the glucose responses to ΔFosB hypothalamic overexpression at an earlier time point after injection (2 weeks), a time at which the energy metabolism and fat effects are not yet manifested. Indeed, 2 weeks post-injection, AAV-ΔFosB showed no change in body weight and visceral fat pad weight from the control (Fig. 4A, B). Likewise, AAV-ΔFosB mice displayed no improved reduction in glucose and insulin levels in the GTT (Fig. 4C,D). At the same time point however, despite no apparent reduction in weight or fat, ITT demonstrated lower glucose levels (Fig. 4E), similar HOMA-IR (Fig. 4F), both indicating initiation of higher insulin sensitivity at this time point. Finally, increased insulin-induced Akt phosphorylation in skeletal muscle was already observed in AAV-ΔFosB mice at this time point (Fig. 4G). Taken together, these results suggest that the increase in insulin sensitivity in AAV-ΔFosB mice is not a secondary effect of the reduction in visceral fat, but rather the result of direct neuronal regulation downstream of hypothalamic ΔFosB.

### ΔFosB exerts its favorable effects on glucose metabolism via AP1 antagonism

**Figure 5:**
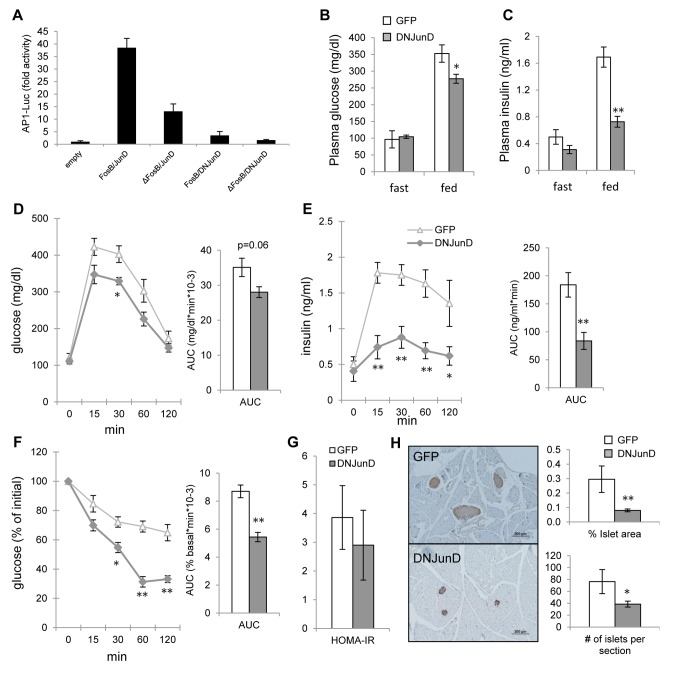
ΔFosB exerts its favorable effects on glucose metabolism via blockade of AP1 Mice were stereotaxically injected into VHT with AAV-DNJunD or AAV-GFP and glucose metabolism was assessed 8 weeks post-surgically (n=4). (**A**) 6X TRE-driven luciferase reporting AP1 transactivation following transfection with FosB and JunD isoforms in mHypoE42 cell line. (**B**) Fasted and fed glucose levels (**C**) Fasted and fed insulin levels (**D**) GTT glucose (**E**) GTT insulin (**F**) ITT glucose (**G**) HOMA-IR. (**H**) Immunostaining of pancreas with anti-insulin antibody. Scale bar; 200μm. Histomorphometry of insulin-stained pancreatic islets. Data are presented as mean ± SEM. *p<0.05, **p<0.01.

AP1 transcriptional activity is dependent upon the heterodimerization between Fos and Jun proteins, with antagonistic properties inferred by ΔFosB [21]. However, ΔFosB acts as a mixed agonist-antagonist of AP1 *in vitro* and *in vivo* [23], making it difficult to know whether its effects are due to one or the other. We have previously shown that AAV-driven hypothalamic overexpression of DNJunD, an artificial pure AP1 antagonist, increases energy expenditure and reduces fat to a similar extent as ΔFosB [20], demonstrating that it is the effects of ΔFosB are due to its antagonism to AP1. We therefore determined whether antagonism of AP1 transcriptional activity in the ventral hypothalamus is responsible for the improved glucose metabolism seen in AAV-ΔFosB mice. First, we performed the AP1 reporter assay of various combinations of FosB and JunD isoforms on 6X TRE-luciferase in the mouse hypothalamic cell line mHypoE42 [24]. When ΔFosB or DNJunD was paired with their active binding partners - FosB or JunD - luciferase activity was decreased (Fig. 5A). ΔFosB/DNJunD heterodimer combination displayed the least reporter activity, confirming that both ΔFosB and DNJunD serve as antagonists to AP1 transactivation in hypothalamic cells. Next, we examined glucose metabolism response in mice carrying stereotaxically delivered AAV-DNJunD in the VHT. Similar to AAV-ΔFosB mice, AAV-DNJunD mice exhibited lower fed glucose and insulin levels, lower glucose levels in response to glucose bolus, despite suppressed insulin response during GTT, and smaller pancreatic islets (Fig. 5B-E,H). While HOMA- IR showed only a trend towards reduced insulin resistance (Fig. 5G), ITT revealed greater insulin sensitivity compared to control (Fig. 5F). Collectively, analysis of VHT DNJunD overexpressing mice demonstrated an improved glucose metabolism profile comparable to the one observed in VHT ΔFosB mice. These results suggest that ΔFosB mediates its metabolic effects via central antagonism of AP1 transactivation.

### AAV-ΔFosB mice are resistant to age-related adiposity and impairment in glucose metabolism

**Figure 6:**
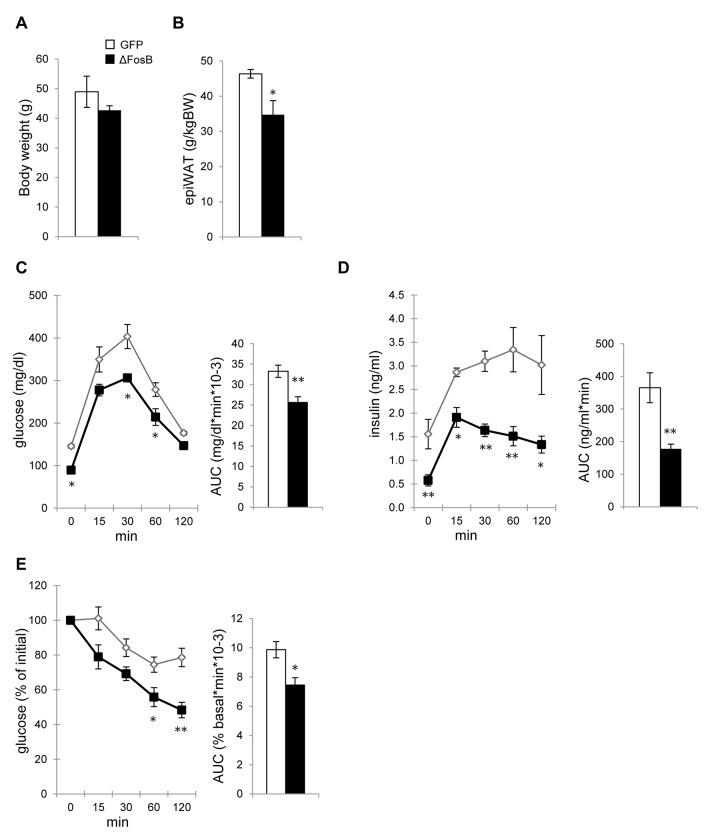
AAV-ΔFosB mice are resistant to age-related adiposity and impairment in glucose metabolism Mice were stereotaxically injected into VHT with AAV-ΔFosB or AAV-GFP and glucose metabolism was assessed 38-40 weeks post-surgically (n=4). (**A**) Body weight (**B**) Abdominal epididymal fat pad weight (**C**) GTT glucose (**D**) GTT insulin. (**E**) ITT glucose. Data are expressed as mean ± SEM. *p<0.05, **p<0.01.

During aging, mammals frequently develop impaired glucose tolerance and insulin resistance [16]. So far our analysis was focused on young animals with normal metabolic profile, yet it was important to delineate whether central ΔFosB could offer protection against vulnerable metabolic conditions, such as those observed with aging. To address this question we performed metabolic analysis on aged mice overexpressing VHT AAV-ΔFosB or AAV-GFP (control) injection, 40 weeks post-surgically, at the age of 48 weeks. Both AAV-ΔFosB and control groups exhibited marked accumulation of body weight and abdominal adiposity, as compared to younger mice (Fig. 6A,B). In contrast, aged AAV-ΔFosB mice showed close to 25% lower abdominal adiposity than AAV-GFP mice (Fig. 6B). GTT analysis revealed that at time zero, aged AAV-GFP mice showed higher insulin levels than their younger AAV-GFP counterparts, 1.56±0.31 ng/mL vs 0.41±0.08 ng/mL respectively (Fig. 6C and Fig. 2D), suggesting the development of age-related hyperinsulinemia. In contrast, the increase in insulinemia in aged AAV-ΔFosB mice was very moderate (0.58±0.12 ng/mL vs 0.19±0.01 ng/mL in younger AAV-ΔFosB mice).

Similarly, aged AAV-ΔFosB mice continued having significantly lower levels of glucose and insulin during the GTT (Fig. 6C,D). Furthermore, the ITT showed that while aged AAV-GFP mice developed insulin resistance compared to younger AAV-GFP mice, as evidenced by slower systemic glucose clearance (Fig. 6E and Fig. 3C), aged AAV-ΔFosB mice maintained higher insulin sensitivity. Taken together, these results indicate that ΔFosB overexpression in the VHT prevents age-related abdominal adiposity and plays a protective role against the glucose intolerance and insulin resistance that develops as a function of age.

### The VHT ΔFosB-mediated improvement in glucose tolerance and insulin sensitivity are dependent on the SNS

**Figure 7:**
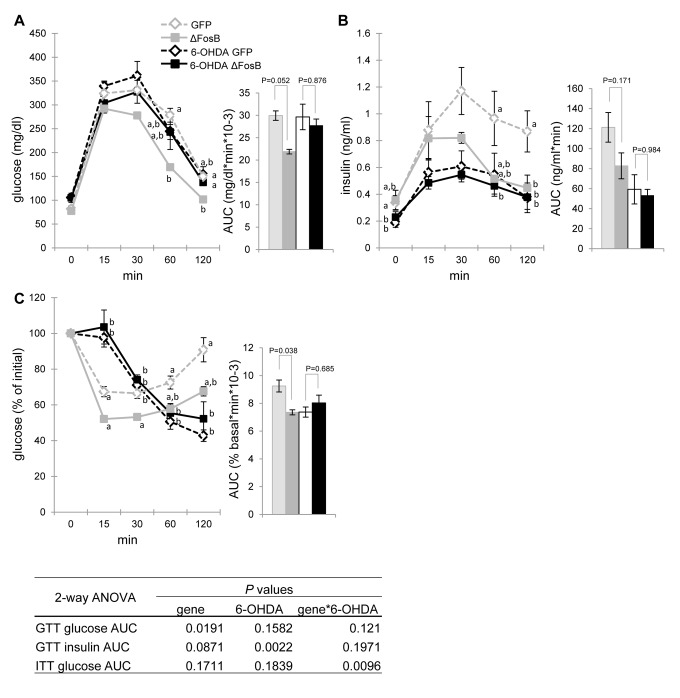
The SNS blockade diminishes the VHT ΔFosB-mediated improvement in glucose tolerance and insulin sensitivity Mice were stereotaxically injected into VHT with AAV-ΔFosB or AAV-GFP, and then were treated biweekly (every two weeks) with SNS blocker 6-OHDA for a total of 3 injections. Metabolic assessment was performed 6-7 weeks post-surgically (n=4-5) (**A**) GTT glucose (**B**) GTT insulin (**C**) ITT glucose. Data are expressed as mean ± SEM. Levels not connected by same letter are significantly different (p<0.05).

As outlined in the introduction, central regulation of glucose and energy metabolism employs both hormonal and neuronal relays. SNS projections linking the hypo-thalamus to the endocrine pancreas are known to directly affect insulin production and secretion [13,14]. We therefore hypothesized that the SNS may be involved in mediating the improvements in glucose metabolism induced by VHT ΔFosB overexpression in older mice. To test this hypothesis, we performed peripheral sympathectomy in AAV-ΔFosB and AAV-GFP (control) mice by administering the catecho-laminergic neurotoxin 6-hydroxydopamine (6-OHDA), which enter cells via monoamine transporters, leading to sympathetic neuronal degeneration [25]. Analysis of glucose metabolism revealed that the SNS neuro-degeneration induced by 6-OHDA completely abolished the reduction in glucose blood levels, despite lower insulin, following GTT in AAV-ΔFosB mice as compared to AAV-GFP control, suggesting that glucose tolerance and insulin sensitivity triggered by central ΔFosB requires an intact SNS (Fig. 7A,B). Moreover, as expected, vehicle-treated AAV-ΔFosB showed increased insulin sensitivity compared to the vehicle-treated AAV-GFP group, whereas 6-OHDA-treated AAV-ΔFosB and AAV-GFP mice had comparable glucose responses to insulin injection (Fig. 7C). Collectively, these data demonstrate the essential role of the SNS in mediating the increase in glucose tolerance and in insulin sensitivity induced by ΔFosB expression in the VHT.

### ΔFosB overexpressed in the VHT regulates insulin secretion and insulin sensitivity via α-, but not β adrenergic receptor in aged mice

**Figure 8:**
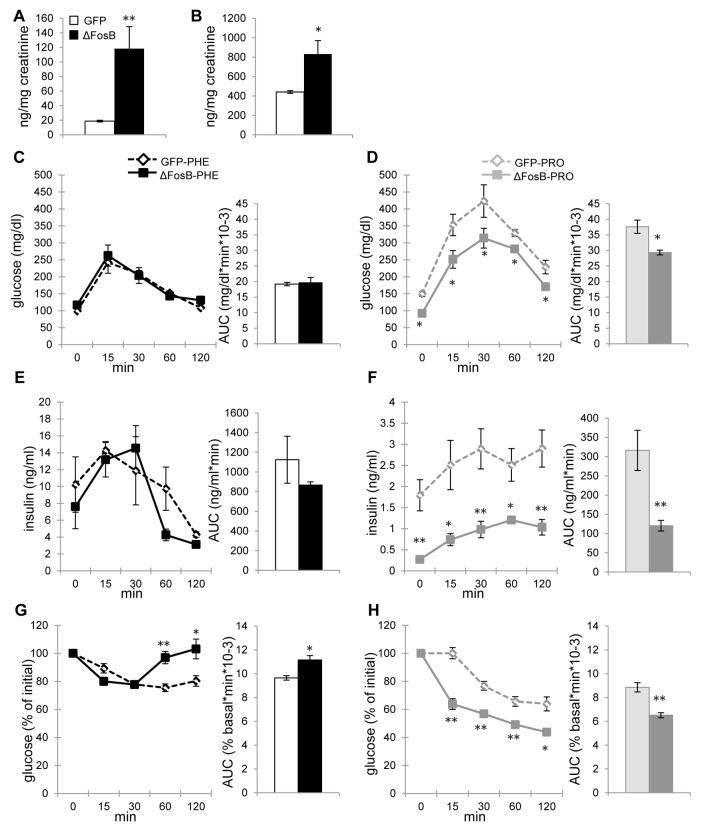
ΔFosB overexpressed in VHT regulates insulin secretion via α-, but not β-adrenergic receptor in aged mice Mice were stereotaxically injected into VHT with AAV-ΔFosB or AAV-GFP and treated 38-40 weeks post-surgically with either α-AR blocker phentolamine (PHE) or β-AR blocker propranolol (PRO) (n=3-4). (**A**) Urinary epinephrine and (**B**) norepinephrine in untreated aged mice. (**C**) GTT glucose of PHE-treated groups (**D**) GTT glucose of PRO-treated groups (**E**) GTT insulin of PHE-treated groups (**F**) GTT insulin of PRO-treated groups (**G**) ITT glucose of PHE-treated groups (**H**) ITT glucose of PRO-treated groups. Data are expressed as mean ± SEM. *p<0.05, **p<0.01.

On the pancreatic α-cells, norepinephrine binds to β2-adrenergic receptor (β2-AR), which stimulates glucagon secretion, whereas on β-cells it binds to α2-AR, which inhibits insulin secretion [13]. Given the essential role of SNS in mediating the metabolic effect of ΔFosB in older mice and the increased epinephrine and norepinephrine levels in aged AAV-ΔFosB mice (Fig. 8A,B), we next attempted to block ARs to determine whether it would have an effect on glucose and insulin profile. Phentolamine and propranolol are commonly used as general α- and β-AR blockers, respectively. Aged AAV-GFP and AAV-ΔFosB mice were pretreated with either agent and subjected to metabolic evaluation. Interestingly, phentolamine-treated AAV-ΔFosB mice showed identical glucose and insulin levels during GTT to phenotolamine-treated AAV-GFP control mice (Fig. 8C,E), suggesting that α-AR activation is essential for conferring the central ΔFosB effect on glucose homeostasis. On the other hand, propranolol-treated AAV-ΔFosB mice maintained their significantly lower glucose and insulin levels compared to AAV-GFP mice (Fig. 8D,F) suggesting that β-AR blockade does not interfere with central ΔFosB action. While increased insulin sensitivity was persistent in propranolol-treated AAV-ΔFosB group, phentolamine-treated AAV-GFP and AAV-ΔFosB groups were both resistant to exogenous insulin probably due to the severe hyper-insulinemia induced by pretreatment of phentolamine (Fig. 8E,G,H). Surprisingly, phentolamine-treated AAV-ΔFosB group showed increased glucose levels at the later time points, 60 and 120 min, possibly suggesting an increased insulin-independent glucose uptake via α-AR in this group. Taken together, α-adrenergic signaling, but not β-adrenergic signaling, plays a critical role in regulating insulin secretion as well as glucose clearance in response to increased expression of ΔFosB in the ventral hypothalamus.

## DISCUSSION

The experimental evidence presented in this paper highlights the role of hypothalamic AP1 trans-criptional machinery in the regulation of glucose homeostasis in both young and aged mice. We have shown that restricted expression of ΔFosB in the VHT, which comprises, among others, ARC and PVN nuclei, exerts beneficial effects not only on bone [17–20], but also on metabolic homeostasis, both in terms of glucose metabolism and adiposity, which persist with aging.

One of the most striking effects of ΔFosB expression in the VHT is the extreme reduction in the size of pancreatic islets, which occurs in parallel with the marked decrease in insulin secretion in response to glucose. This reduction in insulin-producing cells and in insulin secretion could be the result of the marked increase in insulin sensitivity we observed in target organs and/or the result of direct neuronal influences on the pancreas. Our data clearly establish the fact that these changes occur before and independently of the reduction in body fat, which is therefore most likely be the consequence rather than the cause of the improved glucose metabolism. Furthermore, our results establish clearly that the effects of ΔFosB on glucose metabolism are mediated by the SNS, and more specifically by activation of the α-AR pathway, since the effect on glucose handling can be blocked by α-AR, but not β-AR inhibition. Lastly, we show that these ΔFosB-mediated effects render aged mice resistant to age-related adiposity and impairment in glucose metabolism.

Metabolic homeostasis relies on the carefully weighted balance between the blood levels of glucose and insulin, and excess/deficit of either factor may trigger a pathological outcome. For example, if insulin sensitivity is increased without a corresponding correction of insulin release, blood glucose levels remain unbalanced and patients ultimately suffer from weight gain [26]. In similar fashion, advanced type II diabetes is often presented with chronically low insulin secretion on the background of receding insulin sensitivity, resulting in suboptimal clearance of glucose from the blood. Recent genomic analysis in humans and rodents revealed that the gain of function mutation in α2A-AR, is linked to reduced insulin secretion which also occurs in absence of enhanced sensitivity and thus predisposes the development of diabetes [27]. In contrast to the above scenario, in our studies, suppressed insulin secretion did not lead to diabetes and instead, coincided with a robust increase in insulin sensitivity in target organs, which favored the establishment of balanced glucose homeostasis. Since short-term treatment with α-AR blocker phentolamine was sufficient to completely abolish the insulin sensitivity phenotype caused by hypothalamic □FosB, as evident from the ITT, yet was not long enough to affect the islet size, we conclude that the effects on insulin secretion and islet reduction, are secondary to the effects on insulin sensitivity. Our data suggests that central AP-1 blockade triggers an integrative, favorably balanced metabolic response, where activation of α-AR results in higher insulin sensitivity, driving reduction in the size of the pancreatic islets to maintain the balance between the capacity to produce insulin and the reduced need of insulin.

Beyond insulin secretion, α-AR stimulation of adrenomedullary chromaffin cells activates a negative feedback loop, which further limits the release of adrenaline/noradrenaline and thus functions to suppress SNS tone [28–30]. From a clinical perspective, enhanced sympathetic activity coincides with the metabolic syndrome, and on the other hand, weight loss is associated with toning down of the SNS [29]. While the literature strongly supports the inverse relationship between SNS activation and glucose handling, an emerging body of work suggests that SNS innervation of brown fat (BAT) exerts a contrastingly positive effect on glucose handling and energy expenditure [15,31]. Recent studies have shown that BAT control of glucose utilization is orchestrated by SNS-sensitive uncoupling protein 1 (UCP1) and glucose transporter 4 (GLUT4) [32] and this activation is at least in part linked to the activity of hypothalamic AgRP neurons [33].

It has been well recognized that a subgroup of neurons in the hypothalamus are capable of sensing glucose and influencing blood glucose levels by regulating the secretion of anti-insulin hormones and hepatic glucose production [34,35]. The AP1 family member c-fos is a proto-oncogene that is rapidly expressed within some neurons following depolarization, such that c-fos expression is widely used as a marker for neuronal activation [36,37]. This induction of c-fos is also seen in the process of glucose sensing in the hypothalamus. Hypoglycemia or central glucoprivation increases c-fos expression in a number of hypothalamic nuclei, including the ARC, the paraventricular and dorsomedial nuclei and the lateral hypothalamus [38]. In contrast to hypoglycemia, little is known about glucose sensing neurons during hyperglycemia. It is reported that central glucose infusion via the carotid artery increased c-fos expression in ARC and paraventricular nucleus neurons, leading to transient peripheral insulin secretion [39]. It is therefore reasonable to speculate that ΔFosB overexpression in the VHT may play a role in regulating glucose sensing in the VHT neurons and subsequent insulin secretion since ΔFosB is expected to also antagonize c-fos-dependent AP1 signaling and has been shown to desensitize c-fos expression via chromatin remodeling at the c-fos gene promoter [23].

During aging and aging-associated neurodegenerative disorders, the markers of metabolic derangement, such as inflammation and insulin resistance, leading to suppressed glucose uptake, are present both at the periphery [40] and in the CNS neuronal circuits [41,42]. Indeed, the prevalence of diabetes in older adults is more than 25% in the US population and more than twice that of middle-aged adults [43]. Age-related decline in insulin sensitivity has been attributed to various factors including increased abdominal adiposity. The chronic activation of the peripheral SNS has been shown to associate with aging. It is considered to be initiated by increased adiposity and in order to expend excess energy as heat by β-AR thermogenesis. This however eventually evolves into a mechanism that facilitates further development of adiposity by desensitization of β-ARs. Therefore, suppression of age-related adiposity may play a critical role in preventing and/or delaying the progression of metabolic decline with age.

Collectively, our results show that antagonizing AP-1 transcriptional activity by ΔFosB, the naturally occurring splice isoform of FosB, in the VHT leads to a profound improvement in whole body glucose homeostasis via the SNS α-adrenergic pathway. Importantly, these effects persist with aging in mice, conferring protection against the age-related decline in glucose metabolism. Our observations suggest that targeting the AP1 pathway may offer potential means for improvement not only of skeletal homeostasis, but also of whole body glucose homeostasis, preventing not only the development of age-related bone loss but also glucose intolerance.

## METHODS

### Animals

Male C57BL6/J mice were purchased from the Jackson Laboratory and housed in a temperature controlled (25°C) environment under a 12-hour light/dark cycle and fed a rodent chow diet (5058, Pico Lab). All animal protocols were approved by the Harvard University Institutional Animal Care and Use Committee.

### Stereotaxic injections into the VHT

Adeno-associated viruses encoding either ΔFosB-ires-GFP, DNJunD (an N-terminal Δ1–149aa truncation of JunD)-IRES-GFP or ires-GFP alone were injected bilaterally into the ventral hypothalamus of male C57BL/6J mice at 7-8 weeks old weighing 23 - 27g. Stereotaxic coordinates of the injection site were anterior-posterior -2.1 mm, lateral ± 1.3 mm, and dorsal-ventral -5.8 mm at an angle of 10° from Bregma. Animals were analyzed 8-9 weeks after injections, unless noted otherwise.

### Injection validation and analysis of ΔFosB expression

For the purpose of injection validation, saline was injected one side as a sham control and either AAV-GFP or AAV-ΔFosB on the other side of the ventral hypothalamus (Fig. 1A). For further validation of protein expression, whole brain was dissected and separated into three parts: A - anterior part to the coronal cut at the optic chiasm; Hy – hypothalamus; and P - posterior part to the cut at mammillary body; and then subjected to subsequent protein analysis by immunoblotting (Fig. 1B). The hypothalamus was dissected by making two coronal cuts at anterior border of the optic chiasm and the posterior border of the mammillary body, a horizontal cut at the anterior commissure, and two saggital cuts 1.5 mm from the midline.

### Histological analysis of pancreas

Pancreas were collected, fixed overnight in 3.7% PBS-buffered formaldehyde, embedded in paraffin and sectioned at 5 μm. Sections were immunostained for β-cells using rabbit anti-insulin polyclonal antibody (4590, Cell Signaling) and counterstained with hematoxylin. Quantitative histomorphometric analysis of islet area and number was performed using Image J software (National Institute of Health).

### Glucose and insulin tolerance test

Glucose tolerance test (GTT) was performed by administrating glucose (2.0 mg/g BW) intraperitoneally after a 16-hour fast. Blood glucose levels were monitored using glucose test strips and a glucometer (OneTouch ultra, LifeScan) at indicated times. Blood was also collected from tails using EDTA-treated microcapillaries and plasma insulin levels were measured using an EIA kit (ALPCO). For insulin tolerance test (ITT), mice were fasted for 4 hours and injected insulin (1.0 mU/g BW, Lilly) intraperitoneally, and blood glucose levels were measured at indicated times.

### Immunofluorescent staining

Mice were perfused with saline for 10 min and then 3.7% PBS-buffered formaldehyde for 30 min. Whole brains were taken out and post fixed overnight in 3.7% PBS-buffered formaldehyde and then cryoprotected for 36 hours in 20 % sucrose. Brains were then embedded in OCT compound (Tissue Tek), sectioned at 25 μm. ΔFosB immunofluorescence staining was performed with rabbit anti-ΔFosB monoclonal antibody (9890, Cell Signaling) followed by goat anti-rabbit IgG Alexa-fluo-568 conjugated antibody (A11011, Thermo Fischer Scientific). Nuclear staining was done using SlowFade Gold antifade reagent with DAPI (Invitrogen).

### Isolation of primary pancreatic islets

Primary islets were isolated using an intraductal collagenase technique [28]. Briefly, after clamping the common bile duct at its entrance to the duodenum, pancreas was injected with 3 ml of a collagenase P (1mg/ml, Roche) solution in M199 media (Sigma). Dissected pancreas was then digested for 17 min at 37°C, after which they were disrupted by shaking for 30 seconds. Islets were subsequently purified through 100 μm wire mesh and Histopaque 1077 (Sigma) density centrifugation. Islets were cultured for 1-2 hours at 37°C in RPMI 1640 media (Sigma) supplemented with 10% FBS (26140, Life Technologies), 1% Penicillin and Streptomycin (Life Technologies), handpicked under inverted bright-field microscopy, and allowed to recover overnight at 37°C and 5% CO_2_. Islets were serum and glucose starved (2.8 mM glucose RPMI 1640 without FBS) for 2 hours and then stimulated with 2.8 mM and 25 mM glucose for 1 hour.

### AKT phosphorylation

24-hour fasted mice were i.p. injected with insulin (5 mU/g BW, Lilly) or PBS as vehicle control. 15 minutes later, mice were euthanized and skeletal muscle and fat samples were dissected. Samples were then subjected to western blot analysis using rabbit anti-phospho AKT (Ser473) and rabbit anti-pan AKT monoclonal antibodies (4060 and 4691, Cell Signaling).

### Western blotting

Tissue samples were lysed with modified RIPA buffer (50 mM Tris–HCl, pH 7.5, 150 mM NaCl, 1 mM EDTA, 1% Triton-X-100, 0.1% SDS, 1 mM phenylmethylsulfonyl fluoride) supplemented with protease and phosphatase inhibitor cocktails (Roche). Protein concentrations were determined using BCA kit (Thermo Scientific). Protein samples were separated by 4-15% SDS-PAGE and transferred to nitrocellulose membranes using Trans-Blot Turbo transfer system (BioRad). Membranes were then blocked with 5% skim milk in TBS and incubated with primary antibodies overnight at 4°C. Membranes were then washed with TBST and incubated with HRP-linked anti-rabbit IgG (7074, Cell Signaling) for 1 h and washed with TBST. Immunoreactivities were assessed using ECL plus kit (Perkin Elmer). Membranes were stripped using stripping buffer (Clontech) and reused for β-actin detection (4970, Cell Signaling).

### MIN-6 cell culture and in vitro glucose-stimulated insulin secretion test

A mouse pancreatic β cell line, MIN-6 was a kind gift from Dr. Jun-ichi Miyazaki (Osaka University) and Dr. Donald F. Steiner (University of Chicago). MIN-6 cells are derived from mouse insulinoma cells and displays characteristics of pancreatic β-cells, including glucose-stimulated insulin secretion [44]. MIN-6 cells were plated at a density of 2 × 10^5^ cells per well in 12-well plates and maintained in DMEM with 4.5g/L glucose and L-glutamine (D5648, Sigma) supplemented with 3.7g/L sodium bicarbonate, 5μ/L β- mercaptoethanol, 10% Fetal Bovine Serum (FBS, 26140, Life Technologies) and 1% Penicillin Streptomycin (Life Technologies) at 37°C and 5% CO_2_. At 40-50 % confluency, cells were transfected using X-tremeGENE HP (Roche) with either pcDNA3.1 (Life Technologies) as an empty control or pcΔFosB at the amount of 0.5 μg per well. 4 Days after transfection at about 90 % confluency, cells were serum and glucose starved (2.8 mM glucose DMEM without FBS) for 2 hours and then stimulated with 2.8 and 11.2 mM glucose DMEM without FBS for 1 hour. Media from each well was removed and used to determine insulin levels using an EIA kit (ALPCO).

### Urinary catecholamine levels

Urine was collected from conscious mice for 5-7 consecutive days and pooled. Epinephrine and norepinephrine levels were measured using BiCAT kit (ALPCO). Epinephrine and norepinephrine levels were then normalized by urinary creatinine levels measured by an enzymatic colorimetric method (Stanbio).

### Luciferase assays

Embryonic mouse hypothalamic mHypoE 42 cells were plated at a density of 0.5 × 10^5^ cells per well in 12-well plates and maintained in DMEM with 10% FBS (26140, Life Technologies) and 1% Penicillin Streptomycin (Life Technologies) at 37°C and 5% CO_2_. 24 hours after seeding, cells were co-transfected using X-tremeGENE HP (Roche) with a construct encoding 6X-TRE-firefly luciferase reporter (Clontech) and with a construct encoding a JunD isoform (pcJunD or pcDNJunD) and a constract encoding a FosB isoform (pcFosB or pcΔFosB) to measure AP-1 activity. Transfection efficiency was assessed by co-transfecting a TK *Renilla* luciferase construct (Promega). 36 hours after transfection, a dual luciferase assay (Dual-Glo Luciferase Assay System, Promega) was performed according to the manufacturer's instructions.

### Peripheral sympathectomy with 6-OHDA

To induce peripheral sympathectomy, mice received intraperitoneal (i.p.) injections of 6-hydroxydopamine (6-OHDA, Sigma) at doses of 100 mg/kg BW on day -5 and 250 mg/kgBW on day -3. Vehicle group received the solvent, 0.02% ascorbic acid in saline on each day. After stereotaxic surgery to inject AAV-GFP or AAV-ΔFosB in VHT was performed on day 0, mice received biweekly (every two weeks) i.p. injections of 6-OHDA or vehicle. Mice were subjected to metabolic tests after third biweekly i.p. injection (day 42) then euthanized for analysis.

### Adrenergic receptor blockade

To study the effect of α-adrenergic and β-adrenergic signaling on glucose metabolism, mice were pretreated intraperitoneally with 10 mg/kgBW phentolamine (Sigma) and 5 mg/kgBW propranolol (Sigma) 30 minutes before glucose and insulin challenge at GTT and ITT, respectively.

### Statistics

Results are given as mean ± SEM. Statistical analysis were performed using unpaired 2-tailed Student's t test or two-factor ANOVA followed by post hoc test, with p≤0.05.
